# Endothelium-Dependent Induction of Vasculogenic Mimicry in Human Triple-Negative Breast Cancer Cells Is Inhibited by Calcitriol and Curcumin

**DOI:** 10.3390/ijms23147659

**Published:** 2022-07-11

**Authors:** Gabriela Morales-Guadarrama, Edgar A. Méndez-Pérez, Janice García-Quiroz, Euclides Avila, Rocío García-Becerra, Alejandro Zentella-Dehesa, Fernando Larrea, Lorenza Díaz

**Affiliations:** 1 Departamento de Biología de la Reproducción Dr. Carlos Gual Castro, Instituto Nacional de Ciencias Médicas y Nutrición Salvador Zubirán, Vasco de Quiroga No. 15, Belisario Domínguez Sección XVI, Tlalpan, Ciudad de México 14080, Mexico; gabriela.mguadarrama@gmail.com (G.M.-G.); edgar.mendez.p3@gmail.com (E.A.M.-P.); janice.garciaq@incmnsz.mx (J.G.-Q.); euclides.avilac@incmnsz.mx (E.A.); fernando.larreag@incmnsz.mx (F.L.); 2 Programa de Investigación de Cáncer de Mama, Instituto de Investigaciones Biomédicas, Universidad Nacional Autónoma de México, Ciudad de México 04510, Mexico; rocio.garciab@iibiomedicas.unam.mx (R.G.-B.); azentell@iibiomedicas.unam.mx (A.Z.-D.); 3 Departamento de Biología Molecular y Biotecnología, Instituto de Investigaciones Biomédicas, Universidad Nacional Autónoma de México, Ciudad de México 04510, Mexico; 4 Departamento de Medicina Genómica y Toxicología Ambiental, Instituto de Investigaciones Biomédicas, Universidad Nacional Autónoma de México, Ciudad de México 04510, Mexico; 5 Unidad de Bioquímica, Instituto Nacional de Ciencias Médicas y Nutrición Salvador Zubirán (INCMNSZ), Ciudad de México 14080, Mexico

**Keywords:** co-culture, tubulogenesis, vascular mimicry, breast cancer, epithelial-to-mesenchymal transition

## Abstract

In highly aggressive tumors, cancer cells may form channel-like structures through a process known as vasculogenic mimicry (VM). VM is generally associated with metastasis, mesenchymal phenotype, and treatment resistance. VM can be driven by antiangiogenic treatments and/or tumor microenvironment-derived factors, including those from the endothelium. Curcumin, a turmeric product, inhibits VM in some tumors, while calcitriol, the most active vitamin D metabolite, exerts potent antineoplastic effects. However, the effect of these natural products on VM in breast cancer remains unknown. Herein, we studied the effect of both compounds on triple-negative breast cancer (TNBC) VM-capacity in a co-culture model. The process of endothelial cell-induced VM in two human TNBC cell lines was robustly inhibited by calcitriol and partially by curcumin. Calcitriol promoted TNBC cells’ morphological change from spindle-like to cobblestone-shape, while curcumin diminished VM 3D-structure. Notably, the treatments dephosphorylated several active kinases, especially those involved in the PI3K/Akt pathway. In summary, calcitriol and curcumin disrupted endothelium-induced VM in TNBC cells partially by PI3K/Akt inactivation and mesenchymal phenotype inhibition. Our results support the possible use of these natural compounds as adjuvants for VM inactivation in patients with malignant tumors inherently capable of forming VM, or those with antiangiogenic therapy, warranting further in vivo studies.

## 1. Introduction

Triple-negative breast cancer (TNBC), the most aggressive tumor phenotype of the mammary gland, is commonly resistant to conventional therapy, has very limited approved targeted treatments, and is commonly associated with poor prognosis and a high recurrence risk. Among the signaling pathways known to be involved in the malignant features of TNBC tumors, the phosphatidylinositol-3-kinase/Protein kinase B (PI3K/Akt) signaling pathway plays a fundamental oncogenic role in promoting proliferation and survival of cancer cells [1,2]. Notably, the activation of this and other canonical pathways, such as p38/Mitogen-activated protein kinase (MAPK), is critical for vasculogenic mimicry (VM) formation in breast cancer [3,4,5]. VM is the process cancer cells undertake to form tube-like or micro-vessel-like structures in solid tumors, and is clinically relevant given its association with metastasis and resistance to antiangiogenic treatments. The VM vessel network compensates for the lack of endothelial-derived vessels in avascular tumors, allowing access to blood and providing an escape route to invade distant sites. Besides hypoxia, tumor microenvironment-derived factors may act as drivers of VM, including vascular endothelial growth factor (VEGF) and cytokines, such as transforming growth factor beta (TGFB) [5,6,7]. Notably, some of these factors are related to the promotion of epithelial-to-mesenchymal transition (EMT), a process that in breast cancer has been linked with the generation of cancer cells with a stem cell-like phenotype, which are believed to be the source of VM [8]. Due to the intrinsic plasticity of VM-forming cells, they may transdifferentiate into endothelial-like cells, displaying stemness features, high renewal ability, metastatic capacity, and chemoresistance [8,9]. All these properties may contribute to the poor prognosis and compromised therapeutic response of patients with breast tumors undergoing VM [5]. Therefore, implementing VM targeting strategies is of utmost importance, especially for TNBC tumors, in which a significantly higher VM rate has been reported, compared to non-TNBC [10]. Importantly, antiangiogenic treatments can induce VM by generating hypoxia, which is why their combination with anti-VM compounds can help reduce treatment-resistance. 

Regarding VM targeting, the natural compound curcumin, derived from *Curcuma longa*, is known to suppress VM-drivers and VM-formation in different types of cancer cells; however, to date, its effects on VM in breast cancer has remained unexplored [11,12]. The anti-VM potential of curcumin in hepatocellular carcinoma, a tumor characterized by hyper-vascularization and enriched vascular networks, was shown to involve the inhibition of the PI3K/Akt signaling pathway. Likewise, in choroidal melanoma, the curcumin anti-VM effect involved the down-regulation of the EphA2/PI3K/matrix metalloproteinase (MMP) pathway [11,12]. In addition, curcumin has also been shown to inhibit VM in squamous cell carcinoma of the larynx through the inhibition of the Janus kinase-2 (JAK-2)/signal transducer activator of transcription protein (STAT) signaling pathway, and the downregulation of MMP-2 and VEGF expression [13].

On the other hand, calcitriol, the most active vitamin D metabolite, exerts pro-differentiating and antiproliferative effects in breast cancer [14], and has shown an anti-EMT effect in epithelial cells through inhibiting the PI3K/Akt/β-catenin pathway [15]. Also, calcitriol exerts potent anti-inflammatory activity, partially through downregulating JAK-STAT pathway activation and inflammatory cytokine production, processes known to be involved in carcinogenesis and VM [16,17]; however, calcitriol’s effects upon VM has not been explored. Regarding this, while this article was under peer review, Khuloud Bajbouj et al., reported that calcitriol differentially downregulated VM-related pathways and the expression of key VM markers in calcitriol-treated breast cancer cells by an in silico analysis of transcriptomic databases [18]. The same research group also showed that a very high concentration of calcitriol (10 μM) stimulated the expression of tissue inhibitors of MMPs (TIMPs) and decreased that of MMPs, lowering the formation of VM in MDA-MB-231 cells [18]. The effects of curcumin, those of nanomolar calcitriol concentrations, and those exerted by the combination of these compounds on VM development in breast cancer, as well as the mechanisms potentially involved, do, however, remain unknown, which were the originating concepts of our research question. To address this matter, we implemented a model that included the participation of tumor microenvironment-derived factors involved in VM-induction. Knowing that endothelial cells can induce tumor cells to undergo VM [19], we used an in vitro co-culture model of endothelial-induced VM in TNBC cells co-cultured with endothelial cells. Once we characterized the VM-process in our experimental model, we tested the effects of calcitriol, curcumin, and their combination on the ability of tumor cells to form tube-like structures. Furthermore, for mechanistic insights, we explored the ability of the natural compounds to dephosphorylate several kinases known to be involved in VM formation. Part of the novelty of this research resides in the experimental model that we used, the calcitriol concentrations tested that can be safely achieved in patients, and the compounds combinatorial effect upon VM. 

## 2. Results

### 2.1. Calcitriol, Curcumin and Their Combination Inhibited Endothelial-Dependent VM Formation by Breast Cancer Cells

**Figure 1:**
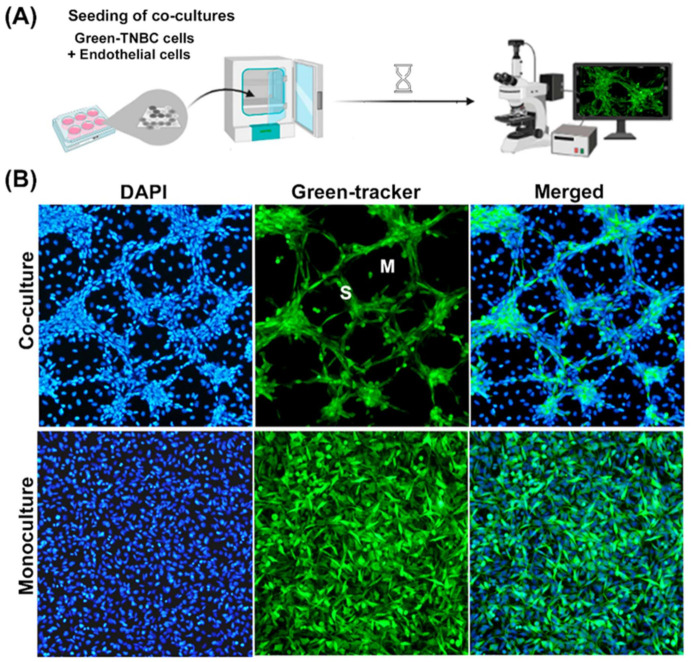
Characterization of the co-culture model of TNBC cells with endothelial cells. (**A**) Illustration of the vasculogenic mimicry process in vitro. TNBC cells stained with a green tracker were seeded with endothelial cells (1:1) and allowed to interact for 2 days. The formation of cord-like networks was evaluated with epifluorescence microscopy. (**B**) MBCDF-T cells stained with a green cell fluorescent tracker were incubated in the presence (upper panels, co-cultures) or the absence (lower panels, monocultures) of unstained endothelial cells (EA.hy926). Afterwards, cells were fixed with ethanol. A mounting medium containing DAPI was used to label cell nuclei in blue (left panels). TNBC cells- vasculogenic mimicry was identified only in co-cultures by the formation of meshes (M) and segments (S) stained in green (upper panels). Merged pictures are shown in right panels. Magnification is 10×.

Paracrine pro-vasculogenic factors from endothelial origin are known to act on tumor cells, altering their behavior [19,20]. As a result of this communication, endothelial cells can enable tumor cells to undergo trans-differentiation, forming cords with a tube-like structure, namely, VM [19,21]. In this study, we used an in vitro co-culture model of endothelial-induced VM in TNBC cells to study the anti-VM effect of calcitriol and curcumin (Figure 1A). As a control, we first validated VM by staining TNBC cells with a green fluorescent Cell Tracker to distinguish which cell type was located in the cord-like tubular formations of the co-cultures. Co-culturing TNBC MBCDF-T cells with EA.hy926 endothelial cells resulted in the development of defined tridimensional interconnecting cord-like tubular networks. These structures were formed by green-stained cancer cells, localized in an elevated focal plane above the endothelial monolayer that seemed to provide the extracellular matrix to support them (Figure 1B, upper panels). Depending on the confluence, the cord-like tubular structures started forming within 24 h of co-culturing, developing meshes containing unstained-endothelial cells underneath, evident only by their blue colored nuclei (Figure 1B). Note that nuclei from endothelial cells in co-cultures appear less bright than those from cancer cells, probably due to the different focal planes and the fact that endothelial cells have the tendency of spreading out. The cells that formed VM in the higher focal plane had a spindle-shaped fibroblast-like phenotype, suggestive of trans-differentiation into a mesenchymal-like lineage (Figure 1B, upper panels). Of note, monocultures of both TNBC cell lines tested in this study could not form VM by themselves, as seen in Figure 1B for MBCDF-T (Figure 1B, lower panels), supporting the pro-VM activity of the endothelial secretome and extracellular matrix.

**Figure 2:**
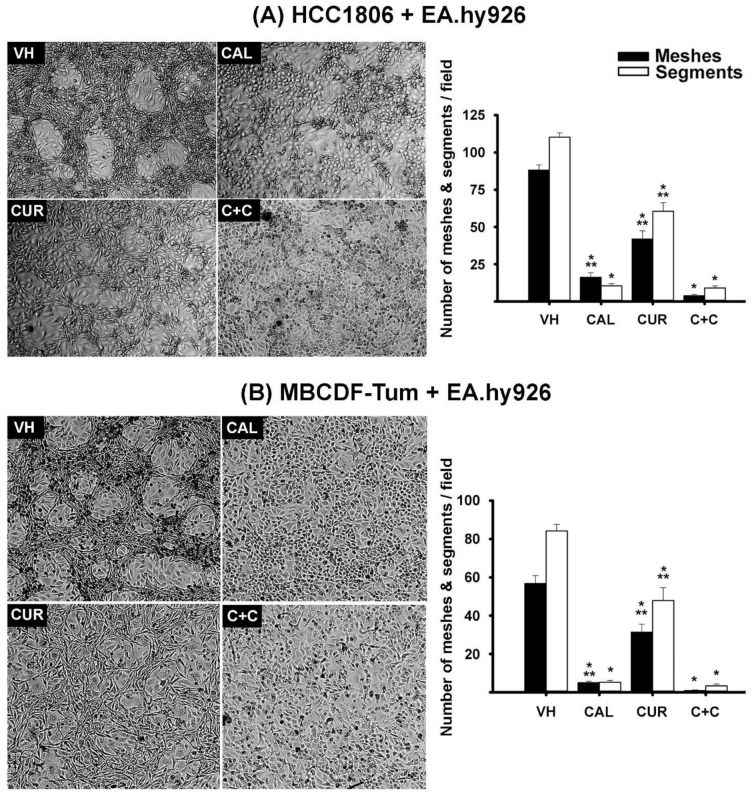
Calcitriol and curcumin inhibited endothelium-induced TNBC-VM capacity in co-cultures. EA.hy926-induced VM was observed in co-cultures of HCC1806 (**A**) and MBCDF-T (**B**) cells incubated with vehicle (VH, ethanol). The presence of calcitriol (CAL, 10 nM) changed cell morphology from spindle-shape to cobblestone-like and abrogated VM formation, while curcumin (CUR, 20 µM) affected the 3D VM structure. The combination of calcitriol with curcumin (C+C) further inhibited VM in both TNBC cells. The number of meshes and segments per visual field were counted by three different observers in hot spots in at least 10 different photographs, considering 3 different experiments for each cell line. The graphics depict the average number of meshes and segments per visual field. * *p* < 0.001 vs. VH. ** *p* < 0.01 vs. C+C. Magnification is 10×.

Interestingly, both TNBC cell lines co-cultured with endothelial EA.hy926 cells and incubated in the presence of calcitriol failed to form VM, and changed their shape from spindle-like to cobblestone form (Figures 2 and S1). This morphological change of TNBC cells was also observed in monocultures (Figure S1), strongly suggesting cell differentiation and EMT inhibition as possible causes of calcitriol-dependent VM deterrence in co-cultures. Curcumin treatment also affected TNBC-VM formation in the co-cultures; however, its effect was less evident compared with that of calcitriol. Actually, curcumin treatment resulted in the partial breakdown of the VM-3D structure, suggesting that this compound affects cell organization/adhesion in the meshes formed by cancer cells (Figure 2). Regarding morphometric parameters, both curcumin and calcitriol significantly reduced the number of segments and meshes compared to controls, as seen in the graphics of Figure 2. In fact, the compounds were able to inhibit VM in a concentration-dependent manner (Figures S2 and S3). Remarkably, in both TNBC cell lines co-cultured with endothelial cells, the combination of calcitriol and curcumin further abrogated VM formation (Figures 2 and S4).

### 2.2. In TNBC Cells Co-Cultured with Endothelial Cells, Calcitriol and Curcumin Inhibited the Phosphorylation of Several Kinases of the MAPK, PI3K/Akt/mTOR, PI3K/Akt/CREB and PI3K/Akt/Gsk Signaling Pathways

**Figure 3:**
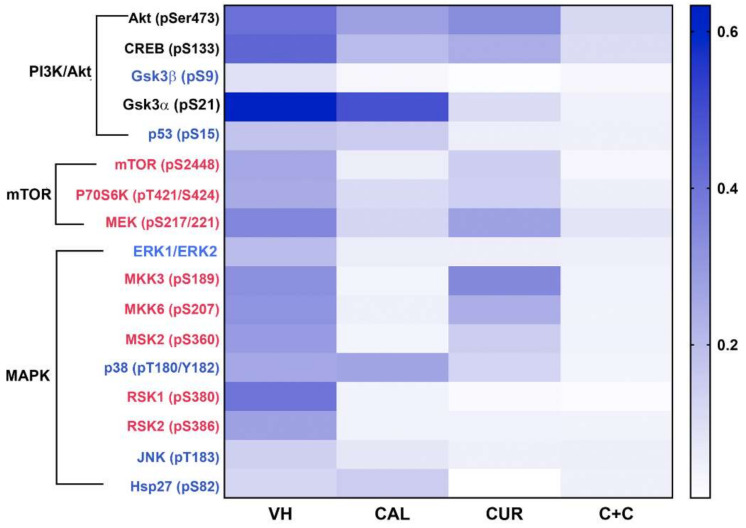
In MBCDF-T/EA.hy926 co-cultures, calcitriol and curcumin inhibited the phosphorylation of several protein targets of the MAPK, PI3K/Akt/mTOR, PI3K/Akt/CREB and PI3K/Akt/Gsk signaling pathways. The phosphorylation profile of kinases involved in signaling pathways relevant to VM was differentially inhibited by calcitriol (CAL, 10 nM), curcumin (CUR, 20 µM) and their combination (C+C). A semi-supervised hierarchical clustering of the targets differentially phosphorylated detected with a multiplex antibody array was carried out and results are shown in the heat map clustered in three main signaling pathways: PI3K/Akt, mTOR and MAPK. The more strongly phosphorylated kinases in vehicle-treated co-cultures (VH) are labeled in black. Kinases mostly inhibited by calcitriol are labeled in red, while phosphorylated protein targets more strongly downregulated by curcumin are labeled in blue. N = 4.

**Figure 4:**
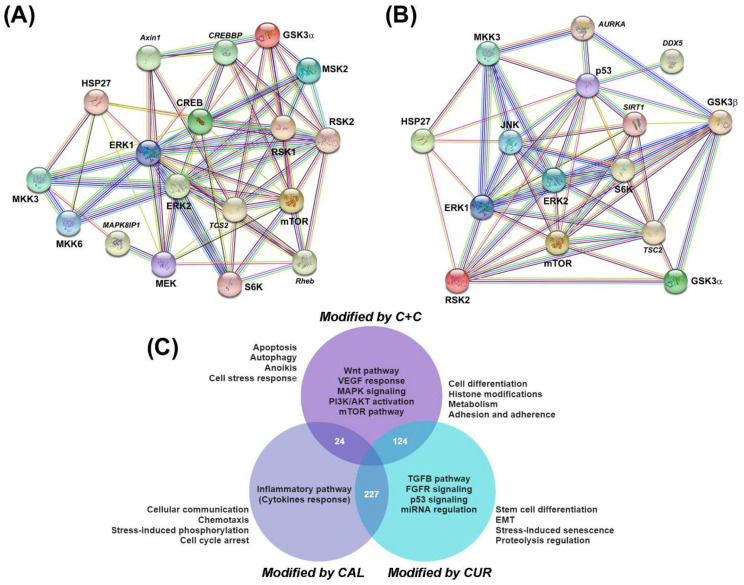
Interaction networks and biological processes regulated by calcitriol, curcumin or their combination involved in the anti-VM effect of the compounds. STRING analysis of deregulated kinases in calcitriol (**A**) or curcumin (**B**) treated co-cultures. Images in (**A**,**B**) show the physical and functional protein interactions. Protein names in italics represent the top predicted interactors of the network. (**C**) Prediction of the biological processes differentially regulated in each treatment group. A total of 227 processes were co-regulated by calcitriol (CAL) and curcumin (CUR); 24 processes were shared by the CAL and CAL+CUR (C+C), and 124 were shared in the CUR and C+C groups. Inside each circle, the main pathways involved are depicted. Outside, near each circle, the biological processes in which protein complexes were predicted to participate are shown.

To study the phosphorylation profile of kinases involved in signaling pathways relevant to VM, we chose MBCDF-T/EA.hy926 co-cultures. A semi-supervised hierarchical clustering of the targets differentially phosphorylated detected with an antibody array (human MAPK Phosphorylation Antibody Array, ab211061, Abcam) was carried out. Results were then represented on a heat map clustered in groups encompassing three main signaling pathways: PI3K/Akt, mammalian target of rapamycin (mTOR), and MAPK (Figure 3). We found that Akt, together with the cAMP response element-binding protein (CREB) and glycogen synthase kinase-3 (Gsk3)α (labeled in black, Figure 3), were the kinases more strongly phosphorylated in control co-cultures. Remarkably, calcitriol or curcumin downregulated the phosphorylation of these proteins as well as p53, P70S6K, and p38, while their combination further inhibited the overall kinases activation status (Figure 3 and Figure S2). On the other hand, the kinases of mTOR complexes and some of the MAPK, such as MKK3, MKK6, MSK2, RSK1 and RSK2 (labeled in red, Figure 3), were strongly inhibited by calcitriol, while the phosphorylation status of Gsk3α, Gsk3β, p53, p38, JNK and Hsp27 (labeled blue, Figure 3) was more intensely downregulated by curcumin. The graphical representation of symbolic kinases inactivated by the compounds with statistical significance is provided in Figure S5. These results suggested that each treatment differentially targeted distinctive parts of the signaling pathways. When combined, a global effect upon dephosphorylation was enhanced, leading to a more potent VM functional inhibition. Furthermore, the STRING analysis indicated that these proteins interacted forming complexes between them, and were involved in several biological functions, as shown in Figure 4A,B. Among predicted processes regulated by calcitriol and curcumin, the VM-related pathways involving Wnt, VEGF, MAPK, PI3K/AKT and mTOR were identified (purple circle, Figure 4C). Interestingly, only calcitriol was related to the inflammatory pathway involving cytokine production and response (navy blue circle, Figure 4C), while curcumin was related to signaling of TGFB, fibroblast growth factor receptor (FGFR), microRNA (miRNA) and p53 (light blue circle, Figure 4C). Moreover, the combination of calcitriol and curcumin affected networks related to cell death, such as apoptosis, anoikis and autophagy, while curcumin alone was associated with senescence and EMT. Finally, major drivers involved in VM are plasticity and adhesion molecules, which were predicted to be affected by both compounds (Figure 4C).

## 3. Discussion

Tumor growth and dissemination significantly depend on forming a functional vascular network. Many mechanisms are involved in this process, but VM stands out among them in the case of highly malignant hypoxic tumors. VM comprises de novo generation of vessels lined with tumor cells, bypassing the lack of functional endothelial vessels. This process can lead to resistance to drugs intended to block tumor vascularization, explaining their failure in the clinic. In this study, we found that the natural compounds calcitriol and curcumin inhibited endothelial-induced VM-formation in TNBC cell lines in vitro, which was significantly potentiated by the concomitant use of both compounds. Calcitriol’s mode of action seemed to involve EMT inhibition, as judged by the change in the cancer cells’ shape from spindle-like to cobblestone, which would explain the ability of this hormone to robustly block VM from the start. Indeed, VM is highly dependent on the mesenchymal cell phenotype and the stemness characteristics of the cells, which confer cell plasticity. In this regard, calcitriol is known to block EMT via the inactivation of PI3K/Akt/β-catenin signaling, and to induce cell differentiation [15]. In addition, calcitriol is a potent anti-inflammatory hormone that inhibits inflammatory cytokine expression, such as interleukin-6 (IL6) [16]. Recently, it has been described that IL6 from endothelial origin acts as a paracrine factor upon MDA-MB-231 breast cancer cells, inducing VM formation [19], warranting further studies on calcitriol effects upon inflammatory cytokines in co-cultures of TNBC and endothelial cells. Altogether, this may help to explain the strong anti-VM effects of calcitriol. Curcumin, on the other hand, did not inhibit EMT in this study, as judged by the lack of modification of the cells’ fusiform shape. As a result, cord like tubular structures were partially formed by curcumin-treated cancer cells. However, in general, these structures lacked the 3D configuration observed in control cultures, and significantly fewer segments and meshes were formed in comparison to vehicle-treated co-cultures of HCC1806. Considering a previous study showing that curcumin induced cell death in endothelial cells, while inhibiting both MBCDF-T and EA.hy926 cell proliferation and migration [22], it is possible that these effects were involved in the anti-VM mechanism of curcumin observed herein, affecting both cell components of the co-culture. In addition, curcumin is known to exert anti-tyrosine kinase activity [23], which is important to the VM process, since some of its drivers, such as VEGF, mediate their effect through tyrosine kinase receptors. Another possibility to explain curcumin’s disruptive effect on the VM 3D structure is the downregulation of intercellular-adhesion molecules required for holding the 3D structure together. In this regard, it has been described that curcumin blocks the cell surface expression of adhesion molecules in endothelial cells, reduces cancer cell binding to extracellular matrix proteins, and significantly decreases the adhesion propensity of breast cancer cells [24,25,26]. These curcumin effects, together with calcitriol pro-differentiating, anti-inflammatory and anti-EMT effects, might explain the ability of both compounds to significantly inhibit VM in our co-cultured TNBC cells. Supporting this, among the predicted processes regulated by curcumin in combination with calcitriol in our STRING analysis were adhesion, cell differentiation and several signaling pathways involved in VM. In this regard, calcitriol and curcumin in combination globally affected the phosphorylation status of a panel of kinases relevant to the VM process. In particular, signaling components of the PI3K/Akt pathway seemed to be the most important for VM formation in our co-culture system, including Akt, CREB and Gsk3α. The latter was in line with known drivers of the VM process that activate PI3K/Akt signaling, including some tumor microenvironment-derived factors such as cytokines, FGF and VEGF [27,28,29,30,31,32]. These and other growth factor-dependent activation of the PI3K/Akt pathway results in Gsk3 inactivation, a process associated with cancer progression. Indeed, Gsk3 activity is known to be inhibited through phosphorylation of Gsk3α at serine 21 and Gsk3β at serine 9. These specific serine residues in Gsk3 are known targets of Akt and other kinases able to phosphorylate them [33]. Loss of Gsk3 activity leads to the blockade of its antineoplastic effects, since Gsk3 acts as a tumor suppressor by negatively regulating molecules involved in cancer development [33]. Remarkably, the phosphorylation of Gsk3α at serine 21 and Gsk3β at serine 9 was found downregulated by curcumin and calcitriol in this study. Since Gsk3 is phosphorylated by Akt, the calcitriol and curcumin-dependent downregulation of Akt phosphorylation found herein was likely the cause of Gsk3 dephosphorylation, partially explaining the anti-VM activity of both compounds. Notably, through docking, it has been described that curcumin fits into the binding pocket of Gsk3β, modulating its activity [34]. Furthermore, curcumin has also been reported to dephosphorylate Akt and Gsk3 in acute lymphoblastic leukemia [35] and to dramatically downregulate the expression of Akt in TNBC cells, resulting in the suppression of cellular proliferation and migration [36]. Similarly, and as found in this study, calcitriol has previously been shown to reduce Akt phosphorylation in breast cancer and renal epithelial cells [15,37].

Noteworthy is the fact that, besides being involved in VM development, the MAPK, PI3K/Akt/mTOR and PI3K/Akt/Gsk signaling pathways are also implicated in different cellular functions relevant to cancer progression, such as cell proliferation, adhesion, migration, metabolism, and survival [4,28]. Therefore, the fact that calcitriol and curcumin significantly downregulated these pathways further supports the antineoplastic potential of these natural compounds alone and combined.

An interesting finding in this study was the dephosphorylation of p53 at serine 15 by curcumin and its combination with calcitriol. Such dephosphorylation is expected be-cause PI3K-related protein kinases ATM and ATR, responsible for the covalent modification on p53, are inhibited by curcumin [38]. Phosphorylation of p53 at serine 15 is required for the activation of p53-responsive promoters [39]. Thus, it seems that curcumin interferes with the p53 transcriptional activity blocking the DNA damage response in cancer cells.

Overall, the data suggest that endothelial cells’ paracrine signaling induced TNBC tumor cells to form VM by activating Akt, CREB and Gsk3α among other important factors related to plasticity and angiogenesis. Calcitriol significantly prevented VM formation in TNBC co-cultures, while curcumin deterred VM 3D structures, at least partially, through downregulating the phosphorylation of several PI3K/Akt pathway components and p53. The combination of calcitriol and curcumin further inhibited the global protein phosphorylation status, explaining the improved effect on VM inhibition. Interestingly, the phosphorylation pattern evoked by the combination of calcitriol and curcumin, is similar to what could be expected when using a multi-kinase inhibitor drug. All considered, calcitriol and curcumin are a good option to use as antiangiogenic therapy adjuvants to help avoid VM generation. Moreover, since these natural compounds’ combination has shown in vivo antiangiogenic effects *per se* [22], the fact that it also exerts anti-VM activity further supports its therapeutic plausibility in a clinical adjuvant context, warranting more studies. In conclusion, our study provides novel knowledge, showing, for the first time, that curcumin and calcitriol alone and combined inhibit VM formation in TNBC cells, through the downregulation of PI3K/Akt signaling.

## 4. Materials and Methods

### 4.1. Cell Cultures and Treatments

In this study we used two human TNBC cell lines: the MBCDF-Tum (MBCDF-T), which was generated from a primary cell culture of a TNBC invasive ductal breast carcinoma [22], and the commercial cell line HCC1806 (ATCC, Manassas, VA, USA). For the co-culture model, we used the human endothelial cell line EA.hy926 (ATCC CRL-292). Both cell lines were maintained under standard culture conditions in control medium (DMEM-F12 medium, 100 units/mL penicillin, 100 µg/mL streptomycin, 5% fetal bovine serum), while experimental procedures were carried out in the same medium, but supplemented with charcoal-stripped fetal bovine serum. Co-cultures were implemented by simultaneously seeding endothelial cells with breast cancer cells (1:1 ratio, 150,000 cells per cell line) on square glass coverslips placed into 6-well plates.

### 4.2. Reagents

The curcumin and calcitriol used for our in vitro studies were from Sigma (Sigma–Aldrich, St Louis, MO, USA). Ethanol (0.1%) was used as a vehicle for both compounds. For characterization of cell co-cultures, TNBC cells were labeled by incubation of live cells with 5-chloromethylfluorescein diacetate, CellTracker Green CMFDA Dye (Invitrogen, Eugene, OR, USA). Cell culture media, antibiotics, and fetal bovine serum were from Gibco/Invitrogen (Invitrogen, CA, USA).

### 4.3. Evaluation of Calcitriol and Curcumin Effects on VM Networks in TNBC Cells

To validate our VM model, TNBC cells were stained with a fluorescent green Cell Tracker™ (ab138891, Abcam, Cambridge, MA, USA) and seeded in monoculture or co-cultured with endothelial cells (1:1). After 2 days, cells were washed and fixed in 80% ethanol for 10 min. After air drying, a drop of UltraCruz™ mounting medium containing 4′,6-diamidino-2-phenylindole (DAPI, Santa Cruz Biotechnology, Santa Cruz, CA, USA) was added to coverslips, which were then placed onto slides and further photographed with a conventional fluorescence microscope equipped with an Olympus DP72 camera (Olympus Optical Co., Ltd., Tokyo, Japan), a 100 W high-pressure mercury burner and suitable filters. 

To quantitatively evaluate the effect of curcumin and calcitriol on the vasculogenic capacity of TNBC cells, the treatments were introduced at the time of co-cultures seeding. Ethanol (0.1%) was used as the vehicle, and different calcitriol and curcumin concentrations were tested, either alone or combined. Co-cultures were visually inspected daily, and after VM formation in vehicle-treated co-cultures, bright field images were acquired in VM-hot spots by conventional microscopy. We chose bright-field images to assess cell morphology, since it yields the possibility to evaluate more descriptive features, such as cell shape and texture in live cells, considering the two cell types of the co-cultures. Experiments were repeated at least three times. Three different observers counted segments and meshes *per* visual field in hot spots of several 4X images. Segments were cords/branches formed by cells, delimited by two junctions, while meshes referred to closed areas surrounded by segments (Figure 1).

### 4.4. MAPK Activation/Phosphorylation Array and Analysis of Protein–Protein Interaction Networks

In order to unveil pathways involved in the inhibitory effect of curcumin and calcitriol on the ability of TNBC cells to form VM, we compared the changes in kinase activation of the MAPK and PI3K/Akt pathways by using the human MAPK Phosphorylation Antibody Array (ab211061, Abcam), which allowed us to simultaneously evaluate several phosphorylated proteins in our co-cultures cell lysates. All steps were carried out as recommended by the manufacturer. The phosphorylation sites on each protein tested were: Akt (pS473), CREB (pS133), ERK1 (pT202/Y204), ERK2 (pT185/Y187), GSK3α (pS21), GSK3β (pS9), HSP27 (pS82), JNK (pT183), MEK (pS217/221), MKK3 (pS189), MKK6 (pS207), MSK2 (pS360), mTOR (pS2448), p38 (pT180/Y182), p53 (pS15), P70S6K (pT421/S424), RSK1 (pS380) and RSK2 (pS386). After treating the co-cultures with the vehicle, calcitriol, curcumin, or their combination, cell lysates were prepared using the provided lysis buffer supplemented with a phosphatase and protease inhibitor cocktail, and stored at −80 °C until assay performance. Total protein in cell lysates was quantified using the BCA Protein Assay kit (Thermo Fisher Scientific, Rockford, IL, USA) and 500 μg were applied to previously blocked membranes and incubated overnight at 4 °C. The membranes were washed and subsequently incubated with HRP-Anti-Rabbit IgG and detection antibody cocktail. Chemiluminescent signals were acquired and analyzed using the ChemiDoc XRS+ System (BioRad). Semi-quantitative comparisons between treatments were performed after normalization against positive controls, which are included in each membrane. Spots with no signal were disregarded. For visual comparison of relative differences among treatments, we categorized the positive results by canonical pathways in a heat map and also used graphic representation for specific phosphorylated kinases using the GraphPad Prisma 7 software. Two different experiments were run in separate membranes. We tested every target in each membrane twice. To construct a functional association network for the proteins involved in the effects of the treatments, targets with fold changes greater than 0.5 were subjected to a prediction analysis for protein-protein interactions by using the Search Tool for the Retrieval of Interacting Genes/Proteins (STRING, https://string-db.org/, last accessed on 25 April 2022), considering no more than 5 predicted interactors for each condition and associated biological processes.

### 4.5. Statistical Analysis

One-way ANOVA followed by the Holm-Sidak method was used for multiple comparisons using a specialized software package (SigmaStat, Jandel Scientific). Data were expressed as the mean ± standard error of the mean (SEM). The differences between calcitriol and calcitriol + curcumin in Figure 2 were analyzed by Student’s *t*-test. The results were considered significant at *p* < 0.05.

## 5. Conclusions

Calcitriol and curcumin suppressed endothelial-induced TNBC cell VM formation, at least in part, by blocking the PI3K/Akt pathway, and subsequently affecting several cellular survival processes. Our results suggest the potential clinical use of calcitriol and curcumin to inhibit VM in TNBC tumors.

## Data Availability

The authors confirm that the data supporting the findings of this study are available within the article [and/or] its Supplementary Materials.

## Supplementary Materials

The following supporting information can be downloaded at: https://www.mdpi.com/article/10.3390/ijms23147659/s1.
