# Supplementary material for: Endothelium-Dependent Induction of Vasculogenic Mimicry in Human Triple-Negative Breast Cancer Cells Is Inhibited by Calcitriol and Curcumin
Source: Int J Mol Sci. 2022 Jul 11;23(14):7659. doi: 10.3390/ijms23147659 (PMC9318499; doi:10.3390/ijms23147659)
Supplement: Supplementary file 1 [file ijms-23-07659-s001.zip › ijms-1768859-supplementary.pdf]

Supplementary Figure S1

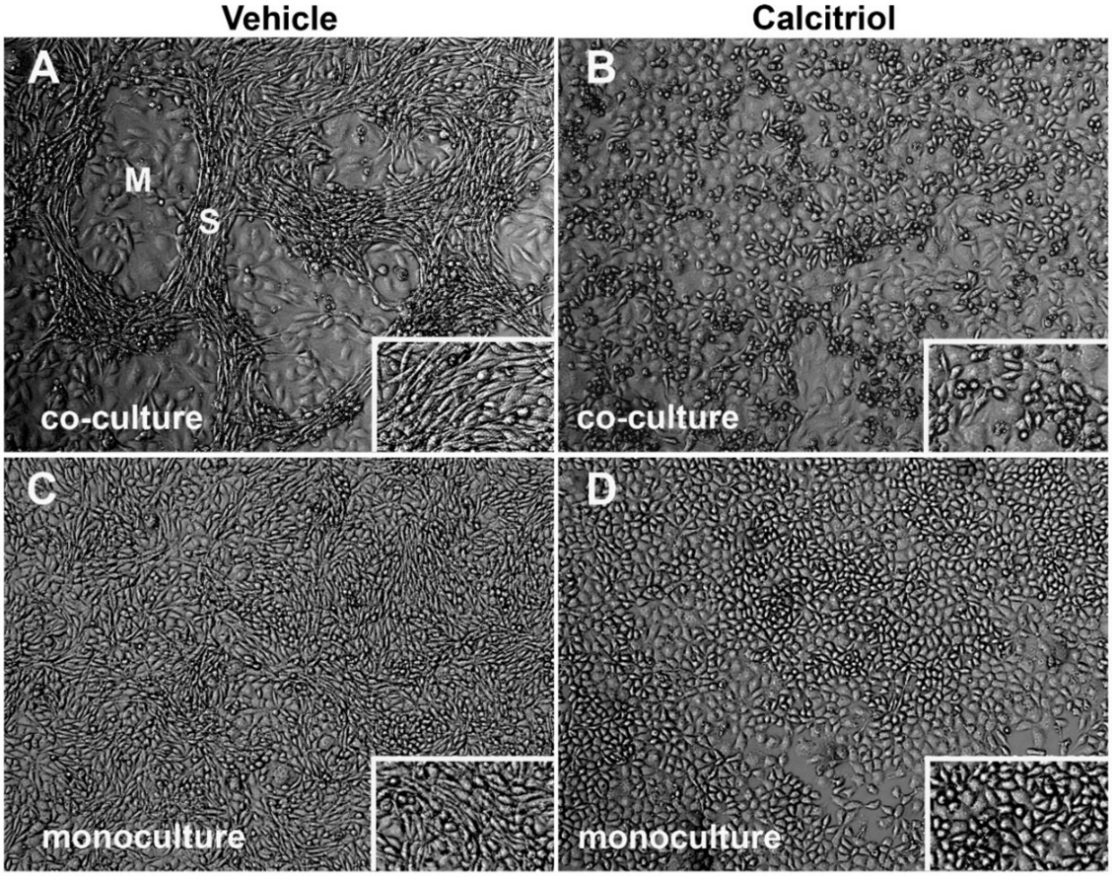

Figure S1. Calcitriol induced a morphological cell change from spindle-like to cobblestone shape in co-cultures and monocultures of TNBC cells. Co-cultures of endothelial cells with TNBC cells (**A**,**B**) or TNBC cells in monoculture (**C**,**D**), were treated with vehicle (**A**,**C**) or 10 nM calcitriol (**B**,**D**) for 2 days. Afterward, bright-field images were acquired by conventional microscopy (10×), and representative images are shown. M and S stand for mesh and segment, respectively, to illustrate these structures. Insets show an amplified part of corresponding image.

Supplementary Figure S2

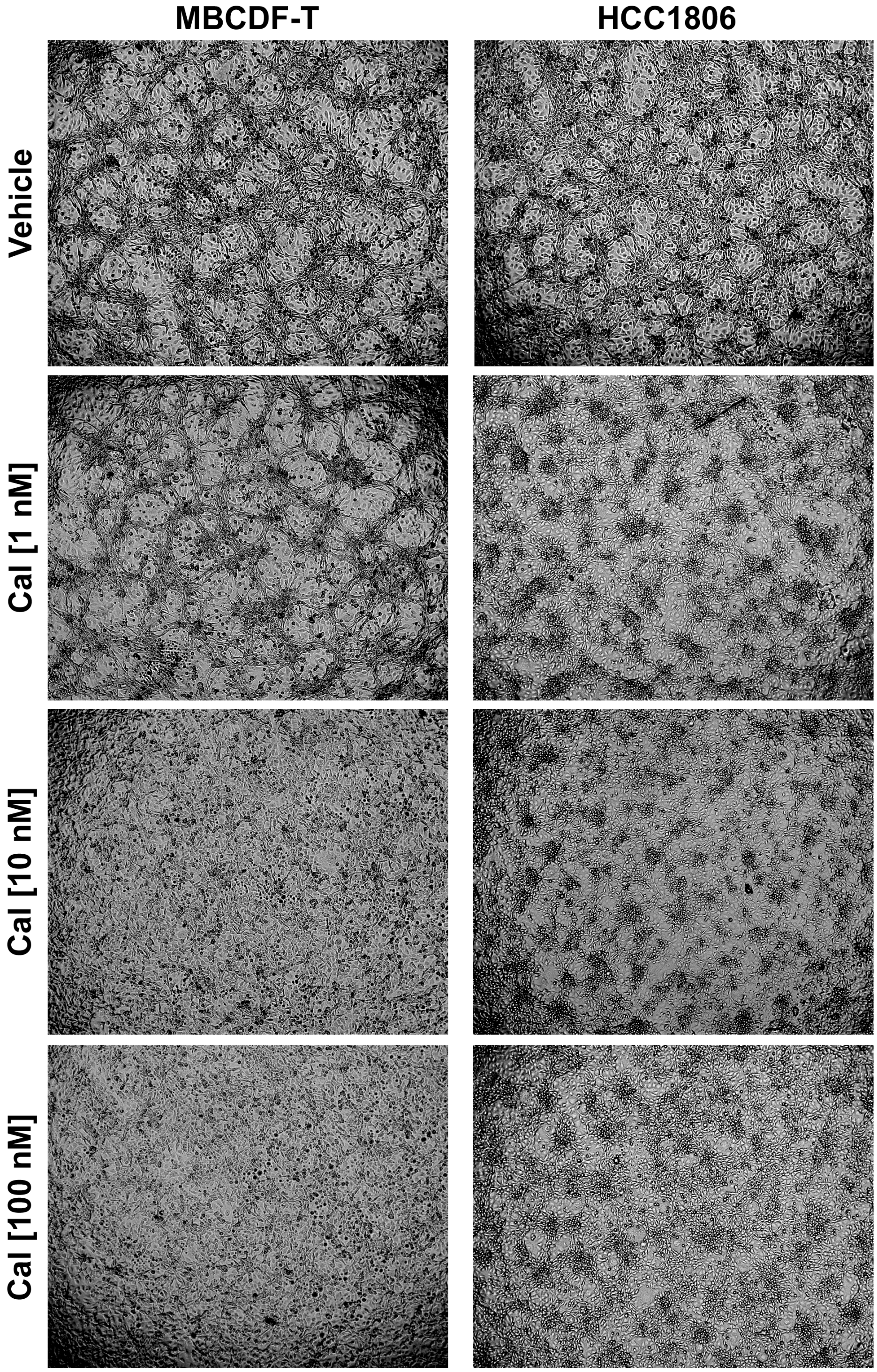

Figure S2. Dose-response anti-VM effect of calcitriol in co-cultures of two different TNBC cells with endothelial cells. Co-cultures of MBCDF-T/EA.hy926 and HCC1806/EA.hy926 were exposed to increasing calcitriol (Cal) concentrations or its vehicle for 2 days, and live cells were photographed at 4× magnification. Representative pictures are shown.

Supplementary Figure S3

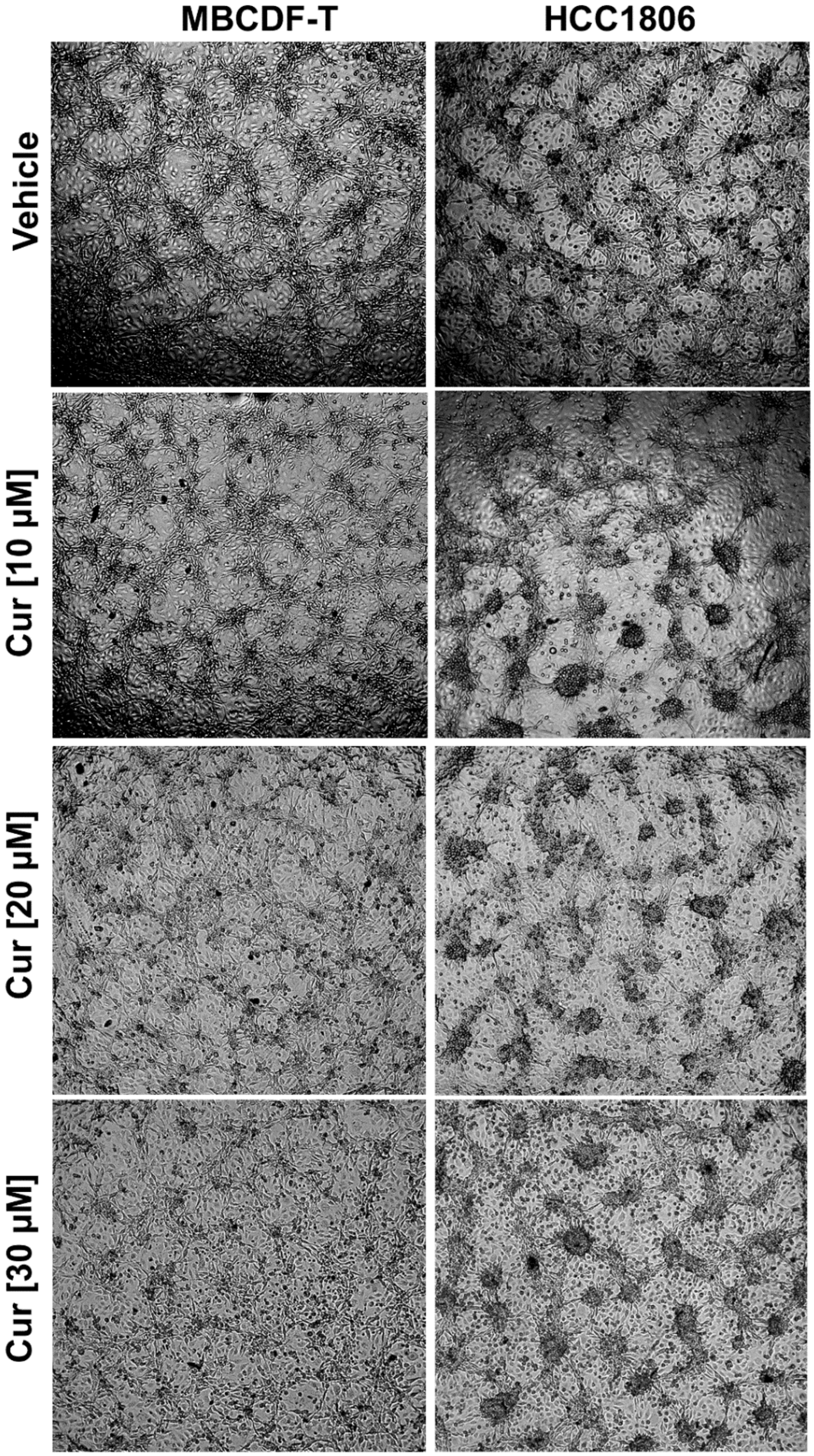

Figure S3. Dose-response anti-VM effect of curcumin in co-cultures of two different TNBC cells with endothelial cells. Co-cultures of MBCDF-T/EA.hy926 and HCC1806/EA.hy926 were exposed to increasing curcumin (Cur) concentrations or its vehicle for 2 days, and live cells were photographed at 4× magnification. Representative pictures are shown.

Supplementary Figure S4

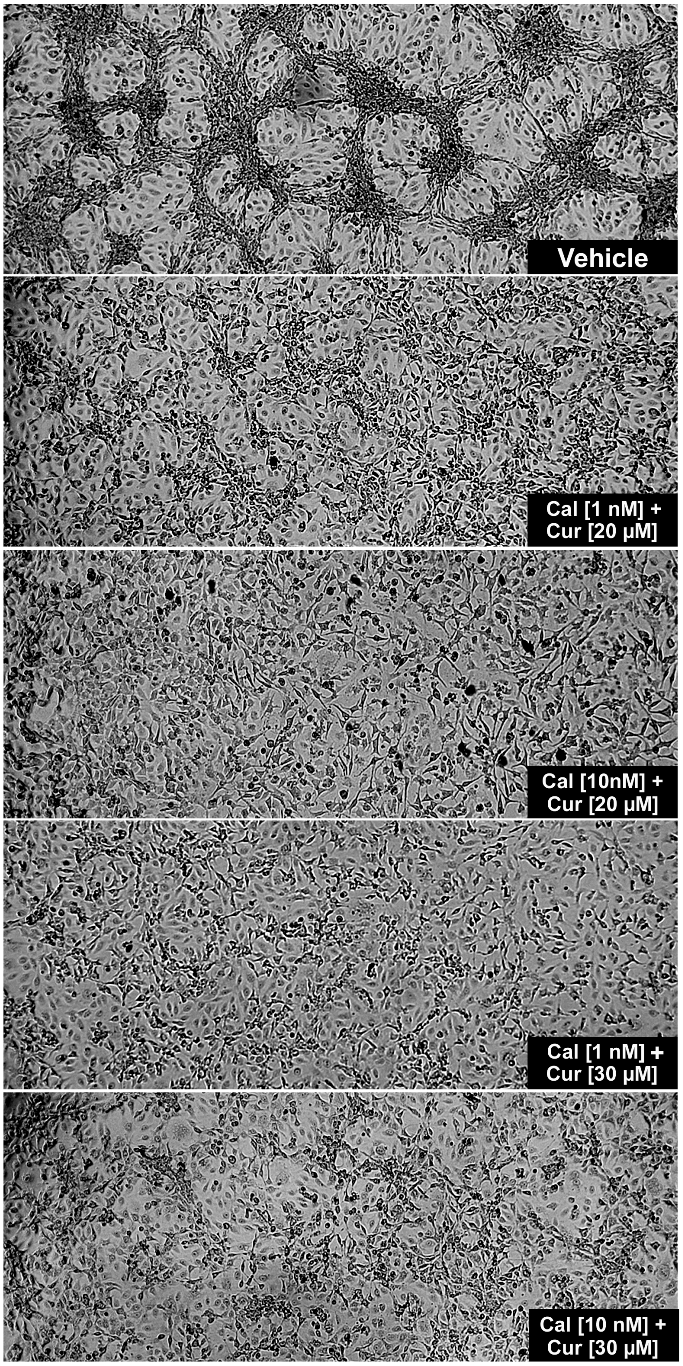

Figure S4. Different combinations of calcitriol and curcumin strongly inhibited VM in MBCDF-T TNBC cells. The anti-VM effect of calcitriol in combination with curcumin in co-cultures of MBCDF-T/EA.hy926 is shown. Co-cultures were exposed to the indicated calcitriol (Cal) and curcumin (Cur) concentrations or their vehicle (0.1% ethanol) for 2 days, and live cells were photographed at 10× magnification. Representative pictures are shown.

Supplementary Figure S5

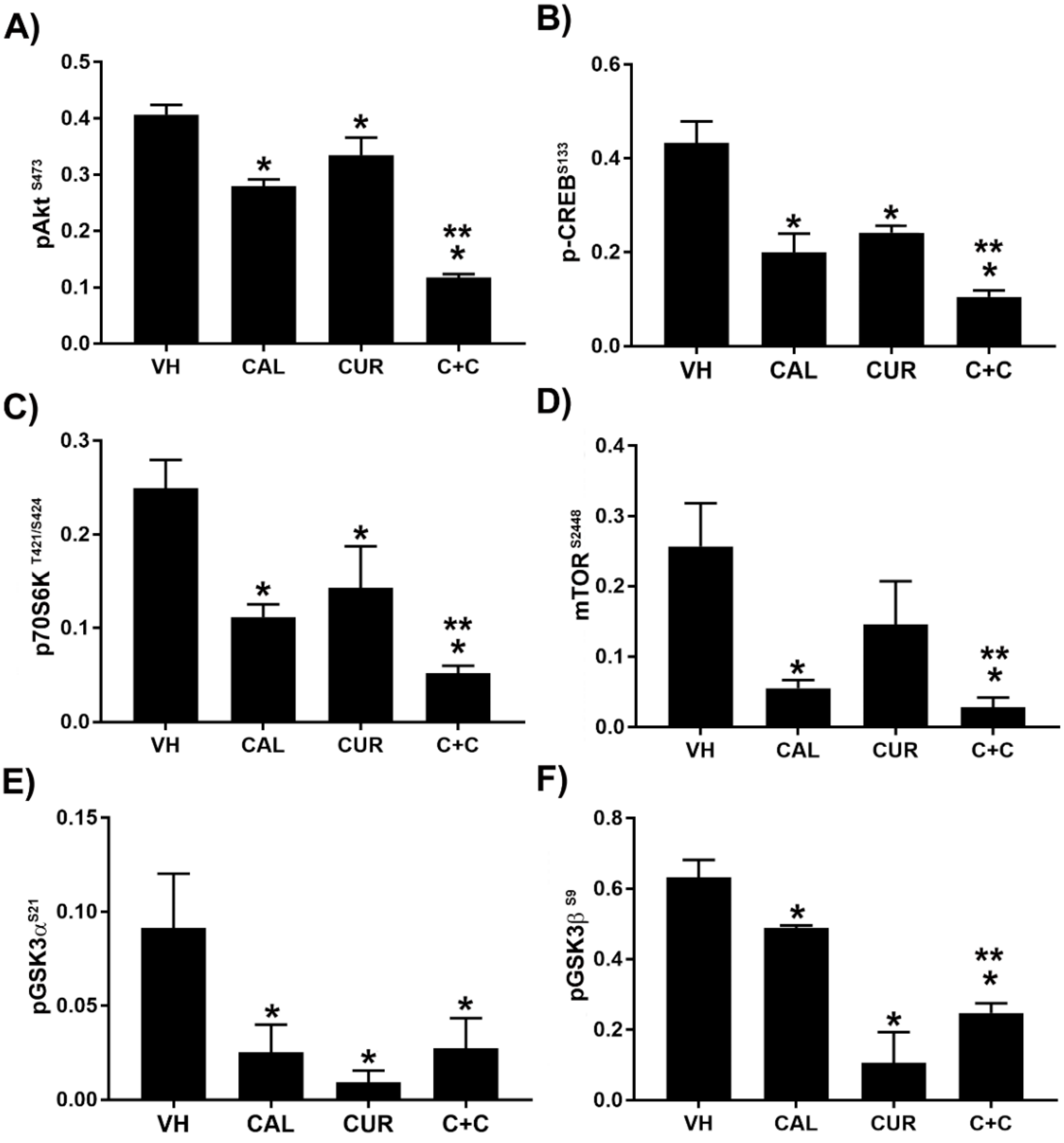

Figure S5. Effect of calcitriol and curcumin treatment on the protein phosphorylation status in endothelial and TNBC co-cultures. Graphical representation of the relative abundance of the phosphorylated kinases in MBCDF-T/EA.hy926 co-cultures exposed to vehicle (VH), calcitriol (CAL, 10 nM), curcumin (CUR, 20 μM) or their combination (C+C) for 24 h. Optical densitometric analysis of selected targets from the multiplex antibody array was analyzed by One way ANOVA. The chosen phosphorylated kinases analyzed were: (**A**) Akt (**B**) CREB, (**C**) p70S6K, (**D**) mTOR, (**E**) Gsk3α and (**F**) Gsk3β. Bars represent mean ± SEM, n = 4. * $p < 0.05$ vs. VH, ** $p < 0.05$ vs. each compound alone.
